# Overall and worst gleason scores are equally good predictors of prostate cancer progression

**DOI:** 10.1186/1471-2490-11-21

**Published:** 2011-10-06

**Authors:** Teemu T Tolonen, Paula M Kujala, Teuvo LJ Tammela, Vilppu J Tuominen, Jorma J Isola, Tapio Visakorpi

**Affiliations:** 1 Institute of Medical Technology, University of Tampere and Tampere University Hospital, Tampere, Finland; 2 Department of Pathology, Centre for Laboratory Medicine, Tampere University Hospital, Tampere, Finland; 3 Department of Urology, University of Tampere and Tampere University Hospital, Tampere, Finland

## Abstract

**Background:**

Gleason scoring has experienced several modifications during the past decade. So far, only one study has compared the prognostic abilities of worst (WGS) and overall (OGS) modified Gleason scores after the ISUP 2005 conference. Prostatic needle biopsies are individually paraffin-embedded in 57% of European pathology laboratories, whereas the rest of laboratories embed multiple (2 - 6) biopsies per one paraffin-block. Differences in the processing method can have a far-reaching effect, because reporting of the Gleason score (GS) is different for individually embedded and pooled biopsies, and GS is one of the most important factors when selecting treatment for patients.

**Methods:**

The study material consisted of needle biopsies from 236 prostate cancer patients that were endocrine-treated in 1999-2003. Biopsies from left side and right side were embedded separately. Haematoxylin-eosin-stained slides were scanned and analyzed on web-based virtual microscopy. Worst and overall Gleason scores were assessed according to the modified Gleason score schema after analyzing each biopsy separately. The compound Gleason scores (CGS) were obtained from the original pathology reports. Two different grade groupings were used: GS 6 or less vs. 7 vs. 8 or above; and GS 7(3 + 4) or less vs. 7(4 + 3) and 8 vs. 9-10. The prognostic ability of the three scoring methods to predict biochemical progression was compared with Kaplan-Meier survival analysis and univariate and multivariate Cox regression analyses.

**Results:**

The median follow-up time of the patients was 64.5 months (range 0-118). The modified GS criteria led to upgrading of the Gleason sums compared to the original CGS from the pathology reports 1999-2003 (mean 7.0 for CGS, 7.5 for OGS, 7.6 for WGS). In 43 cases WGS was > OGS. In a univariate analysis the relative risks were 2.1 (95%-confidence interval 1.8-2.4) for CGS, 2.5 (2.1-2.8) for OGS, and 2.6 (2.2-2.9) for WGS. In a multivariate analysis, OGS was the only independent prognostic factor.

**Conclusions:**

All of the three Gleason scoring methods are strong predictors of biochemical recurrence. The use of modified Gleason scoring leads to upgrading of GS, but also improves the prognostic value of the scoring. No significant prognostic differences between OGS and WGS could be shown, which may relate to the apparent narrowing of the GS scale from 2-10 to 5-10 due to the recent modifications.

## Background

Grading of prostatic needle biopsies has undergone several refinements in the last decade. First, Epstein suggested that a diagnosis of Gleason score (GS) 2 + 2 = 4 cancer should not be made on the needle biopsies, because subsequent radical specimens showed upgrading in virtually all cases [1]. Next, worst Gleason score (WGS) was shown superior to overall Gleason score (OGS) in predicting the final GS of the radical specimen, yielding fewer cases of unwanted upgrading events [2]. The third major adaptation was made in the consensus conference of International Society of Urological Pathology 2005, leading to a refinement called modified GS [3]. In that scheme, any aggressive cancer seen on the needle biopsies should be recorded and incorporated to the GS, even if present in small amount.

Worst Gleason score (WGS) is recommended for individually processed biopsies by ISUP 2005 consensus conference [3]. In the case of pooled biopsies, the exact number of biopsies is sometimes difficult to know due to tissue fragmentation and/or overlapping of the biopsies, and thus, WGS cannot be reliably assessed [3].

According to a recent survey among European pathology laboratories, approximately one half of the participants use individually processed biopsies, while the others immerse multiple biopsies per formalin container without special identification tags (Lars Egevad, personal communication). Individually processed biopsies allow clinicians to localize the histopathological findings to the anatomic biopsy site. In addition, when the biopsy cores are individually embedded in paraffin blocks, a separate GS can be assessed for each biopsy, and the worst of them is usually reported to the clinicians to guide the treatment. Instead, the uropathologists did not reach consensus whether to use worst or overall GS in the case when multiple cancer-containing biopsies are pooled to one formalin container without identification tags [3].

A few studies comparing OGS and WGS have been published and only one of them after the ISUP conference [4]. In three studies WGS at any biopsy site was better than OGS at predicting the pathological T-stage and GS in radical prostatovesiculectomy specimens [2,4,5] whereas in one study, OGS performed better in predicting progression-free survival in patients treated with radiotherapy [6].

Our earlier study analyzing biopsies from endocrine-treated patients indicated that OGS was the strongest independent prognosticator of all histopathological parameters [7]. Gleason score assessment according to ISUP 2005, using the most aggressive pattern as a secondary Gleason grade even when it is present in only a small area, yielded the best prognostic classification using groupings < 7(4 + 3), 7(4 + 3)-8, and 9-10. In the present study, we examined whether the WGS in a single biopsy core would improve prognostic accuracy when compared with OGS. We also evaluated the prognostic value of compound Gleason score from the original pathology reports before the ISUP 2005 era.

## Methods

### Material

The study was approved by the Ethical Committee of Tampere University Hospital (TAUH) and the National Authority for Medicolegal Affairs. From 1999 to 2003, 295 consecutive new prostate cancer patients, diagnosed from core biopsies, were primarily hormonally treated in the TAUH. Representative formalin-fixed, paraffin-embedded samples were available from 236 (80%) cases. Of these, clinical follow-up data were available for 233/236 (99%) cases. The end-point, biochemical progression, was defined as a ≥ 25% rise in PSA, with a PSA value ≥ 2.0 ng/ml above the nadir in two consecutive measurements, as recommended by The Prostate Cancer Clinical Trials Working Group (PCWG2) guidelines [8]. The median PSA value at the time of diagnosis was 15.5 ng/ml (mean 144 ng/ml, S.D. 772). Tumors were organ-confined (clinical T1-2) in 126 patients and advanced (cT3-4) in 107 patients. Bone scintigraphy was done in all symptomatic patients and in asymptomatic patients when PSA was ≥ 20 ng/ml or they had aggressive (original compound GS > 7) prostate cancer. Based on bone scintigraphy, metastasis was detected in 40 (17%) patients. The primary hormonal treatments were luteinizing-hormone releasing-hormone (LHRH) analog (n = 169), surgical castration (n = 43), antiandrogen bicalutamide (n = 21), and maximal androgen blockade (n = 3).

Two slides from each patient were analyzed. The most representative hematoxylin and eosin (H&E)-stained slide, consisting of biopsies from the left or right lobe, was selected and scanned with Aperio ScanScope^® ^XT (software version 9; Aperio Technologies, USA) and viewed in JPEG2000 format using JVSview virtual microscopy software (version 1.2) [9].

The WGS and OGS were evaluated according to the recommendations of the International Society of Urological Pathologists 2005 by one pathologist (T.T.T.) [3]. The evaluation was performed on the scanned images of the most representative side of the prostate on a virtual microscope. The overall Gleason score was derived as a sum of the predominant and the most aggressive (or secondary) patterns of all the biopsy cores, treated as one long core. The worst Gleason score in a single biopsy core was assessed in cases for which one biopsy contained a higher Gleason grade (e.g., 4 + 4 cancer) and other cores a lower grade (e.g. 3 + 4). In the cases in which all positive biopsy cores contained same Gleason grade (e.g., 3 + 3) or there was only one core positive for cancer, the WGS was equal to the OGS. A Gleason score of 7 was considered as two separate grades (e.g., the WGS could equal 4 + 3 and the OGS 3 + 4). The evaluation of CGS was originally made by several pathologists, mainly by two uropathologists, who assessed CGS as sum of the predominant and the second most common Gleason patterns based on the evaluation of needle biopsy specimens from both lobes. In this study the CGS was obtained directly from the original pathology reports.

Two different grade groupings were used: GS 6 or less vs. 7 vs. 8 or above; and GS 7(3 + 4) or less vs. 7(4 + 3) and 8 vs. 9-10.

### Statistical analysis

The agreement between Gleason scoring methods was analyzed with the κ-coefficient method. A survival analysis with PSA progression as end-point was performed using the Kaplan-Meier method, and the statistical significance of survival differences between patient groups was determined with a Mantel-Cox test. The univariate and multivariate Cox regression analyses were performed to calculate the relative risk estimates (RR) and to evaluate the independence of the prognostic grading methods. No clinicopathologic data other than the different Gleason scoring methods were included in the multivariate analysis. However, these data had been analyzed by us previously [7].

## Results

### Basic characteristics

The median age of the patients was 73.8 years (range 52.7-88.8). The median PSA at the time of diagnosis was 15.7 ng/ml (range 2.4-10750.0 ng/ml). The median follow-up time was 64.5 months (range 0-118). The distribution of numbers of biopsy cores per side is provided in Table 1. The differences in progression-free survival between different treatment forms were not assessed because patients were not randomized, and due to bias that LHRH analogue was used in 72.5% of cases.

**Table 1:** Distribution of needle biopsy cores

No. cores/lobe^1^	No. cases (*n *= 236)
1	2
2	7
3	52
4	60
5	50
6	56
7	7
8	1
9	1

^1^The average number of cores per lobe 4.5 (median 4, range 1-9).

### Needle biopsy findings

**Figure 1:**
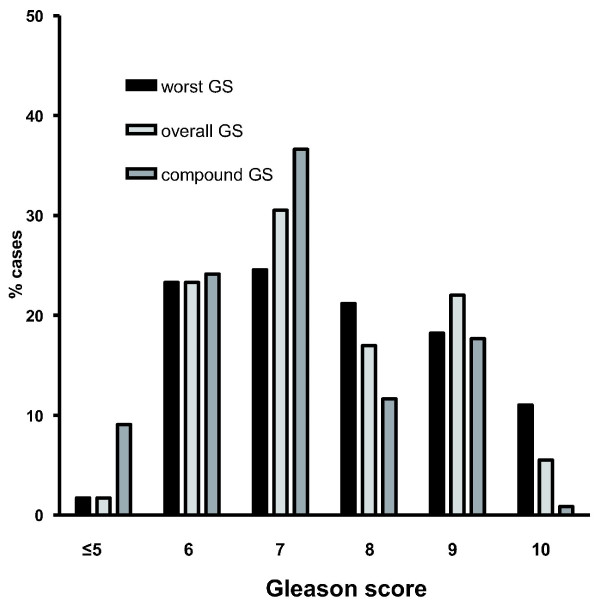
**Gleason score distributions**. Distribution of Gleason scores (GS) according to the grading method. The number of cases with GS 7 is overemphasized by using compound GS, before the revised guidelines by ISUP 2005 were in routine use. Major changes between overall Gleason score (OGS) and worst Gleason score (WGS) are noted in shift from OGS 7 to WGS 8 and from OGS 9 to WGS 10.

The average number of positive biopsy sites was 3.1 (median 3, range 1-7). The number of cases with multiple positive biopsy sites was 191/236 (80.9%). Worst GS was higher than OGS in 43/236 (18.2%) cases. In general, the modified GS system yielded higher Gleason scores. The average GS was 7.6 (median 8, 95%-confidence interval 5.0-10.3) for WGS, 7.5 (7.0, 5.0-10.0) for OGS, and 7.0 (7, 4.5-9.6) for CGS. The distribution of Gleason scores according to grading method is shown in Figure 1. The number of cases with OGS = 7 was 65. In 14 (22%) cases of them there was at least one positive biopsy core containing higher-grade cancer (WGS 4 + 4 = 8). In 12 (31%) of 39 cases with OGS 3 + 4 = 7, a positive biopsy core with the highest score showed WGS 4 + 3. Overall GS = 9 was encountered in 52 cases of which the biopsy core with highest GS showed WGS = 10 in 10 (19%) cases. In three cases the difference between WGS and OGS was 2; in all of them OGS = 8 (3 + 5 or 5 + 3) and WGS = 10 (5 + 5).

### Statistical analyses

**Figure 2:**
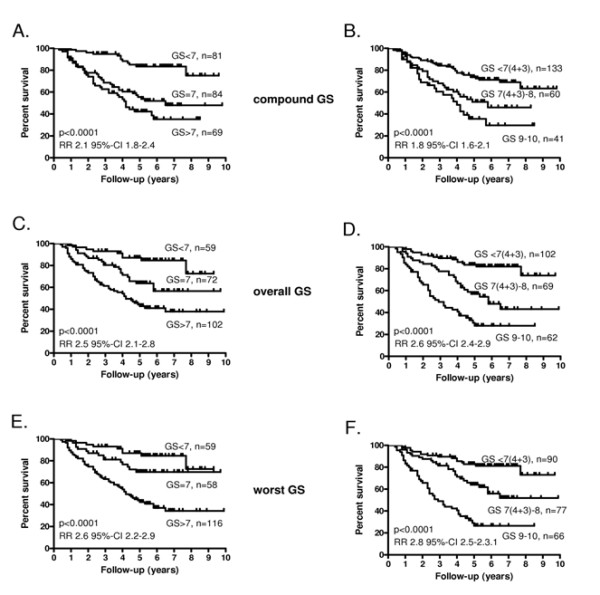
**Survival curves**. Kaplan-Meier progression-free survival curves according to compound Gleason score (CGS) < 7, 7, > 7 from both lobes (A), CGS < 7(4 + 3), 7(4 + 3)-8, 9-10 from both lobes (B), overall Gleason score (OGS) < 7, 7, > 7 from the most representative lobe (C), OGS < 7(4 + 3), 7(4 + 3)-8, 9-10 from the most representative lobe (D), the worst Gleason score (WGS) < 7, 7, > 7 in a single biopsy from the most representative lobe (E), the WGS < 7(4 + 3), 7(4 + 3)-8, 9-10 in a single biopsy from the most representative lobe (F). Relative risks (RR) with 95%-confidence intervals (95%-CI) according to Cox univariate analysis as well as p-values according to Mantel-Cox tests are shown.

The agreement between WGS and OGS was high (κ-coefficient = 0.82). A significantly lower concordance was found between WGS and CGS (κ = 0.48) and OGS and CGS (κ = 0.44). All Gleason scoring methods provided prognostically highly significant information (Figure 2A, B, C, D, E, and 2F).

The univariate analyses of OGS and WGS yielded similar relative risks (Figure 2). Re-classification of the Gleason score groups to < 7(4 + 3), 7(4 + 3)-8, 9-10 improved slightly prognostic value of the scoring. In the multivariate analysis of the six different Gleason grading methods, OGS reclassified as < 7(4 + 3), 7(4 + 3)-8, 9-10 was the strongest (and only) independent prognostic factor (RR 2.6, 95% confidence interval 2.0-3.5).

## Discussion

The refinements of the ISUP 2005 consensus conference on Gleason scoring of needle biopsies have generally yielded better prognostic accuracy [10]. Our results indicate that modified Gleason scores according to the ISUP 2005 system are higher than compound GS's from 1999-2003, and this upgrading is associated with improved prognostic accuracy. Moreover, the results suggest, that OGS may be a slightly stronger or at least equally adequate predictor of PSA progression than WGS, when assessed from pooled biopsies.

A major implication of the revised 2005 ISUP guidelines has been the mandate to integrate the most aggressive tertiary patterns to secondary in needle biopsy scoring, even when the pattern is limited to a small area. A recent webmicroscope-based concordance study about Gleason grading of GS 6-8 by the European Network of Uropathologists suggested that general pathologists are starting to overgrade the experts (Lars Egevad, personal communication). Because Gleason grading is subjective, it is not difficult to detect some glandular fusion, and to interpret them as secondary Gleason pattern 4. Due to aforementioned issues, a fraction of cancers previously graded as GS 3 + 3 = 6 would nowadays end up with GS 3 + 4 = 7. Thus, it has been suggested that changing definitions shift the cut-off between low-grade and high grade cancers from 3 + 4 to 4 + 3 [11,12]. The results of the present study are consistent with that.

According to the 2005 ISUP consensus conference, the highest (worst) GS should not be assessed from biopsies immersed in the same formalin container ("pooled biopsies") due to tissue fragmentation [3]. When all six biopsies from one lobe are formalin-fixed in the same container, they may become fragmented or overlap when embedded, disturbing the attempt to assess the WGS of the individual needle biopsies. To avoid this, some laboratories choose to ink pooled cores different colors and thus be more specific about sites and fragmentation. On the other hand, WGS was recently shown to be a better predictor of the histopathological findings from subsequent radical prostatectomy specimens [4]. In our study, the WGS was assessed from the needle biopsies of one prostate lobe embedded in one paraffin block. Because of this, it is possible that our WGS results were biased by tissue fragmentation. However, in the majority of the cases (*n *= 193/236, 82%), the WGS was equal to the OGS. If there were a bias due to fragmentation, we should expect more cases with WGS > OGS.

A major problem when multiple biopsies are stored in one container is that the exact locus information of the biopsies is lost unless site identifiers are used. Another disadvantage is that it is harder to keep all the biopsies in the same plane of section, but this can be avoided in eg. by using foam plastic inside the cassettes. The locus information is essential when considering targeted brachytherapy or cryotherapy in focal carcinomas. Moreover, the anatomic localization of carcinoma foci is useful when planning nerve-sparing radical prostatectomy and to avoid side effects from external-beam radiotherapy. The problems associated with placing multiple biopsies in one container can be overcome by immersing one core biopsy per formalin-container, which is quite laborious for all the participants: the urologist, laboratory technicians, and pathologist. Two major advantages of embedding multiple needle biopsy cores in one paraffin block are the reduced workload and the ability to analyze immunohistochemical stainings from all the biopsies at once, when deemed necessary.

There are a few limitations in the present study. First, although PSA progression works as a surrogate end-point for progressive prostate cancer, it does not necessarily correlate specifically with cancer or overall survival. Due to the small number of deaths in our series, we cannot conclude that OGS was a better prognostic factor in terms of death as a hard end-point. To address this question, a longer follow-up is needed. Second, CGS was not re-evaluated in the present study; instead it was obtained from the original pathology reports, which limits the value of this comparison. Third, the study contained a limited number of cores and the number of cases in which WGS > OGS was rather low (n = 43). Therefore, it is not surprising that the WGS and OGS yield similar results.

## Conclusions

Overall and worst Gleason scores provide comparable prognostic information. We conclude that clinicopathological practice using one container per lobe (six biopsies) and yielding an overall Gleason score is a straightforward and cost-effective procedure that correlates well to prognosis in hormone-treated patients. Therefore, the use of individually embedded biopsies should be dictated by the need for anatomic site information and weighed against the increased workload for the pathology laboratory.

## Competing interests

The authors declare that they have no competing interests.

## Authors' contributions

This study has been designed by PK, TV, and TTT. The needle biopsy samples have been analyzed by TTT. Manuscript has been written by TTT, PK and JI. VT is responsible for the digitalization of the images and virtual microscope system. TLJT has acquired the clinical database of the patients. TV is responsible for the statistical analyses and finalization of the manuscript. Conclusions have been drawn mainly by TTT, JI, TV and PK. All authors read and approved the final manuscript.

## Pre-publication history

The pre-publication history for this paper can be accessed here:

http://www.biomedcentral.com/1471-2490/11/21/prepub

## References

[B1] Epstein JI Gleason Score 2-4 Adenocarcinoma of the Prostate on Needle Biopsy: A Diagnosis That Should Not Be Made Am J Surg Pathol 2000 24 477 478 10.1097/00000478-200004000-00001 10757394

[B2] Kunz GM Jr Epstein JI Should each core with prostate cancer be assigned a separate Gleason score? Hum Pathol 2003 34 911 914 10.1016/S0046-8177(03)00338-1 14562287

[B3] Epstein JI Allsbrook WC Jr Amin MB Egevad L The ISUP Grading Committee The 2005 International Society of Urological Pathology (ISUP) Consensus Conference on Gleason Grading of Prostatic Carcinoma Am J Surg Pathol 2005 29 228 242 10.1097/01.pas.0000173646.99337.b1 16096414

[B4] Kunju LP Daignault S Wei JT Shah RB Multiple prostate cancer cores with different Gleason grades submitted in the same specimen container without specific site designation: should each core be assigned an individual Gleason score? Hum Pathol 2009 40 558 564 10.1016/j.humpath.2008.07.020 19144380

[B5] Poulos CK Daggy JK Cheng L Preoperative prediction of Gleason grade in radical prostatectomy specimens: the influence of different Gleason grades from multiple positive biopsy sites Mod Pathol 2005 18 228 234 10.1038/modpathol.3800302 15475927

[B6] Forman JD DeYoung C Tekyi-Mensah S Bolton S Grignon D The prognostic significance of the worst vs. overall gleason score in patients with multiple positive prostate needle biopsies Int J Radiat Oncol Biol Phys 2000 48 3 suppl 206

[B7] Tolonen TT Tammela TLJ Kujala PM Tuominen VJ Isola J Visakorpi T Histopathological variables and biomarkers enhancer of zeste homologue 2, Ki-67 and minichromosome maintenance protein 7 as prognosticators in primarily endocrine-treated prostate cancer BJU Int http://dx.doi.org/10.1111/j.1464-410X.2011.10253.x 10.1111/j.1464-410X.2011.10253.x 21592298

[B8] Scher HI Halabi S Tannock I Morris M Sternberg CN Carducci MA Eisenberger MA Higano C Bubley GJ Dreicer R Petrylak D Kantoff P Basch E Kelly WK Figg WD Small EJ Beer TM Wilding G Martin A Hussain M Prostate Cancer Clinical Trials Working Group Design and end points of clinical trials for patients with progressive prostate cancer and castrate levels of testosterone: recommendations of the Prostate Cancer Clinical Trials Working Group J Clin Oncol 2008 26 1148 1159 10.1200/JCO.2007.12.4487 18309951 PMC4010133

[B9] Tuominen V Isola J The application of JPEG2000 in virtual microscopy J Digit Imaging 2009 22 250 258 10.1007/s10278-007-9090-z 17999112 PMC3043697

[B10] Billis A Guimaraes MS Freitas LL Meirelles L Magna LA Ferreira U The impact of the 2005 international society of urological pathology consensus conference on standard Gleason grading of prostatic carcinoma in needle biopsies J Urol 2008 180 548 552 10.1016/j.juro.2008.04.018 18550106

[B11] Lotan TL Epstein JI Clinical applications of changing definitions within the Gleason grading system Nat Rev Urol 2010 7 136 142 10.1038/nrurol.2010.9 20157302

[B12] Thompson IM Canby-Hagino E Lucia MS Stage migration and grade inflation in prostate cancer: Will Rogers meets Garrison Keillor J Natl Cancer Inst 2005 97 1236 1237 10.1093/jnci/dji286 16145036

